# Brachio-cephalic ('Gracz') fistula use for continuous hemofiltration in a hemodynamically unstable hemodialysis patient without venous vascular access: a case report

**DOI:** 10.1186/1752-1947-1-39

**Published:** 2007-06-30

**Authors:** Peter E Spronk, Jos NM Barendregt, Guus Crooijmans, Yolande M Vermeeren, Johannes H Rommes

**Affiliations:** 1 Department of Intensive Care Medicine, Gelre Hospitals, Apeldoorn, The Netherlands; 2 Department of Intensive Care Medicine, Academic Medical Center, Amsterdam, The Netherlands; 3 Department of Internal Medicine, Gelre Hospitals, Apeldoorn, The Netherlands; 4 Hermes critical care group, Amsterdam, The Netherlands

## Abstract

Even in patients with chronic renal failure and chronic intermittent hemodialysis, continuous venovenous hemofiltration (CVVH) is the most often practiced renal replacement technique in the intensive care unit. Although patients show less hemodynamic instability during CVVH than during hemodialysis, it requires a blood flow exceeding 200 ml/min in the extracorporeal circuit necessitating the use of large bore catheters. Vascular access in critically ill septic and edematous patients is sometimes difficult, or even impossible.

We describe a technique of using a brachio-cephalic arterio-venous fistula in a hemodialysis patient for continuous hemofiltration (HF) resulting in improved hemodynamic stability.

## Background

Even in patients with chronic renal failure and chronic intermittent hemodialysis (CIHD), continuous venovenous hemofiltration (CVVH) is the most often practiced renal replacement technique in the intensive care unit (ICU) with a filtration rate of at least 2 liters/hour [1]. Patients show less hemodynamic instability during CVVH than during hemodialysis (HD) [2]. However, CVVH requires a blood flow exceeding 200 ml/min in the extracorporeal circuit implicating the use of large bore catheters. Obtaining or maintaining vascular access in critically ill septic and edematous patients is sometimes difficult, or even impossible.

We describe a technique of using a brachio-cephalic (BC) arterio-venous fistula in a hemodialysis patient for continuous hemofiltration resulting in improved hemodynamic stability.

## Case presentation

A 58 year old caucasian male with renal insufficiency due to nephrosclerosis was admitted to the ICU with septic shock following bowel perforation. Previous renal replacement therapies had consisted of, in chronological order, continuous ambulatory peritoneal dialysis (CAPD) for 5 years ending with catheter removal due to bacterial peritonitis. Intermittent HD was then performed on central venous hemodialysis catheters, complicated by bilateral jugular thrombosis. Due to vessel usability, a classical Cimino fistula could not be constructed in the lower arm. Hence, a left-sided BC-fistula was constructed in the upper arm and successfully used for 1 year after which CAPD was resumed for 1 year. No signs of steel syndrome with hand ischemia occurred during the HD period [3]. The catheter was again removed when treatment was complicated by ultrafiltration (UF) failure and bacterial peritonitis. A history of massive vomiting, an abdominal CT-scan and pathological examination of a peritoneal biopsy taken upon removal of the catheter led to a diagnosis of encapsulating peritoneal fibrosis. Treatment for this disorder had been started with prednisolone and tamoxifen while intermittent HD was resumed on the well developed BC-fistula. Abdominal symptoms had practically been absent in the preceding two years.

Due to septic shock and hemodynamic instability, central venous access was needed for CVVH and vasopressor support besides other intravenous administrations. Because the left arm and subclavian vein were left untouched to spare the AV-fistula for future hemodialysis, CVVH was initiated on the left femoral vein while vasoactive medication was given on a right sided subclavian catheter. In spite of prophylactic administration of subcutaneous nadroparine 2850 IU daily, the patient developed a deep vein thrombosis of the left leg 1 week after admission, probably related to the large bore catheter for CVVH. Ultrasound examination confirmed the presence of a thrombus in the left common iliac vein. Further, intermittent fever and a purulent exit-site made the diagnosis of suspected catheter sepsis, after which the left femoral catheter was removed.

Frequent hemodialysis was performed but was complicated by hypotension in spite of the use of vasopressor support and this therapy failed to reverse the severe edematous state. In view of this clinical dilemma, we decided to try hemofiltration by vascular access to the existing brachio-cephalic fistula.

## Hemofiltration

**Figure 1:**
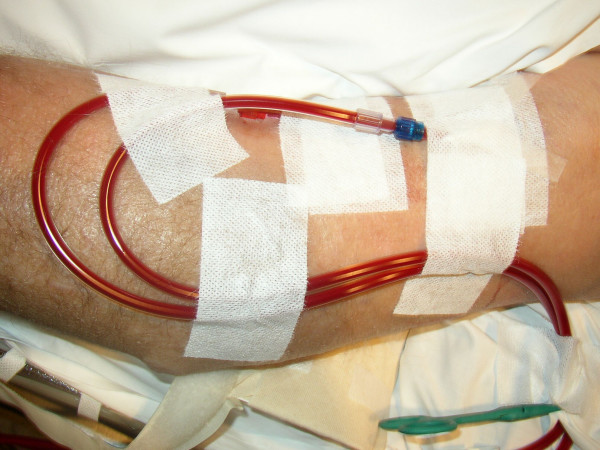
large bore venflon catheters introduced into the arteriovenous fistula.

**Figure 2:**
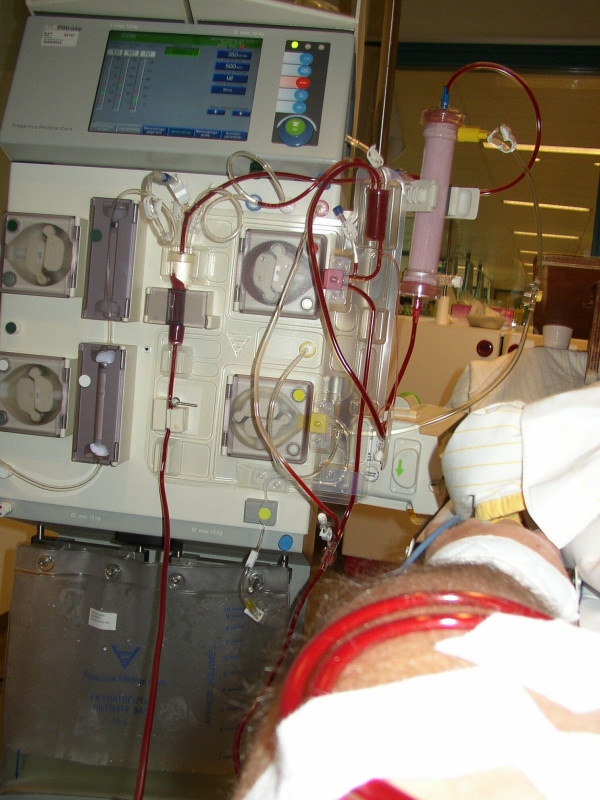
patient connected to hemofiltration machine.

His shunt had been used for 3 years three times a week for intermittant HD without problems. Two large bore teflon canulas (15 Gz, Clampcath, Togo Medikit CO, Japan) were used to access the shunt for connection to the hemofiltration machine (Multifiltrate, Fresenius Medical Care, Germany). The clinical setting is depicted in figure 1 and 2. Subsequently, the normal CVVH protocol was used. Blood flow was started at 200 ml/min without volume extraction, substitution rate was set at 3000 ml/hour. Anticoagulation was done systemically with unfractionated heparin aiming at an activated partial partial prothrombin time between 70–90 seconds in view of the thrombosis. Since hemodynamic parameters remained stable, blood flow was increased to 350 ml/min, substitution rate to 4500 ml/hour, while fluid extraction was started as well. In the subsequent hours, UF rate could be increased to 500 ml/hour without changes in hemodynamic measurements. After 12 hours of continuous hemofiltration, the canulas were removed to spare the AV fistula. In the following 14 days, the patient underwent daily continuous HF with an average volume extraction of 4.5 liters/day. In the mean time, his hemodynamic state had improved in such a way that we successfully switched to intermittent HD.

## Discussion

We describe the use of a brachio-cephalic fistula for HF-treatment in a hemodynamically unstable patient with inaccessibility to the central venous compartment. In critically ill patients, the clinical application of continuous techniques like arteriovenous hemofiltration (CAVH) and intermittent HF have changed treatment modalities of renal failure which used to include only HD. To perform such treatments a reliable vascular access is of vital importance. Unfortunately, multiple vascular access problems are frequently seen among chronic HD or HF patients despite the reliability of the conventional arteriovenous fistula [4]. Since the introduction of large-bore catheters for acute HD, many problems with handling, material, and contamination of these catheters have been described. Nevertheless, catheterization of the femoral and jugular veins with a large-bore catheter has proved to be suitable as a rapid connection process for hemodialysis, hemofiltration, hemoperfusion, and plasmapheresis. In our patient, the femoral catheter had only been in place for 5 days, but thrombosis developed despite thrombosis prophylaxis. Prolonged femoral vein catheterization is a known risk factor of both the femoral and iliac veins thrombosis and stenosis [5]. Although a rising number of ICUs will use extra corporal citrate anticoagulation in unstable patients at risk for bleeding, many dialysis centers still use unfractionated heparine in those cases. The controle of heparinization (usually 2 × normal value of aPTT) and reversibility of heparine overdose (protamine sulfate) are important potential advantages. Moreover, heparine given by the dialysis line may produce a higher local (femoral vein) heparine concentration with concurrent improved prevention of femoral/iliac vein thrombosis when compared to subcutaneous application of low molecular weight heparin analogues. Besides side-effects related to central venous catheters [6], obtaining or maintaining vascular access for continuous hemofiltration can sometimes be problematic, especially in the child or adult in multiple organ failure with edema and/or coagulopathy [7]. Ultrasound guidance for cannulation of the internal jugular and subclavian veins may be used. Nevertheless, common access problems include obstruction of the femoral, subclavian, or jugular veins due to previous thrombosis, insertion difficulties, safety concerns when cannulating the subclavian vein in coagulopathy, and catheter and circuit occlusion due to disseminated intravascular coagulation.

Alarabi *et al*. describe a needleless prosthetic vascular access device (Hemasite) as an alternative solution in patients with high incidence of previous access failures, i.e. 1–8 failures per patient [8]. The 1 year cumulative survival rate of the device was 55%, failure being caused predominantly by thrombosis. However, due to the clinical setting of our patient, this was not an option. One possible alternative to the chosen access strategy in this patient might have been the lumbar insertion of a vena cava catheter [9]. In our centre we have no practice with inserting and using these catheters and the use of such a catheter just proximal to an established venous thrombosis did not seem safe.

There are several risks in using the hemodialysis access for continuous renal replacement. Sparing the access site for future hemodialysis is a well recognized adagium in these patients. Severe bleeding from the access site can occur after needle displacement. Meticulous fixation of the needles and blood-lines was applied to prevent this complication. In spite of these measures we refrained from continuing treatment during nightly hours. However, this approach could have reduced the risk of damaging the access by frequent puncturing. Damaging by frequent puncturing would especially be a problem in a synthetic graft shunt. Infection of the access site however would still be a major concern in any prolonged treatment. Nevertheless, fistula needle insertion for 12 hours seems to be safe. The Tassin experience showed that long term hemodialysis sessions up to 10 hours with fistula cannulation did not produce additional problems with fistulas [10,11].

## Conclusion

While, for obvious reasons, we do not recommend the routine use of existing hemodialysis access for continuous treatment, it is important to realize that when no alternatives are feasible, like in our patient, the hemodialysis access can be used for delivering continuous or semi-continuous hemofiltration therapy in the intensive care unit.

## Competing interests

The author(s) declare that they have no competing interests.

## Authors' contributions

All authors contributed substantially to the manuscript. PES was involved in the primary care of the patient, conceived of the study, contributed to the interpretation and analysis of the data, and drafted the manuscript. JNMB revised the manuscript for intellectual content. GC was involved in the primary care of the patient and contributed to the interpretation of the data. YMV revised the manuscript for intellectual content. JHR conceived of the study, contributed to its design and the interpretation of the data, and revised the manuscript for important intellectual content. All authors approved the final version submitted for publication.
